# Long-Term Sewage Survey of SARS-CoV-2, Influenza A and Respiratory Syncytial Virus (RSV), and Correlation to Human Cases in a City with One Million Inhabitants

**DOI:** 10.3390/microorganisms13102268

**Published:** 2025-09-27

**Authors:** Nathalie Wurtz, Lea Maggiore, Céline Boschi, Alexandre Annessi, Franck Berges, Alexandre Lacoste, Herve Chaudet, Philippe Colson, Bernard La Scola, Sarah Aherfi

**Affiliations:** 1 Microbes, Evolution, Phylogeny and Infections (MEPHI), Aix-Marseille Université, Assistance Publique Hôpitaux de Marseille (AP-HM), IHU Méditerranée Infection, 13005 Marseille, France; nathalie.wurtz@univ-amu.fr (N.W.); celine.boschi@ap-hm.fr (C.B.); herve.chaudet@gmail.com (H.C.); philippe.colson@ap-hm.fr (P.C.); 2 Bataillon de Marins-Pompiers de Marseille, 13003 Marseille, France; lea.maggiore@bmpm.gouv.fr (L.M.); alexandre.annessi@bmpm.gouv.fr (A.A.); franck.berges@bmpm.gouv.fr (F.B.); alexandre.lacoste@bmpm.gouv.fr (A.L.)

**Keywords:** wastewater, SARS-CoV-2, influenza virus, respiratory syncytial virus, wastewater-based epidemiology, viral loads

## Abstract

Wastewater-based epidemiology is a robust, scalable, cost-effective, and high-performing tool to monitor and predict SARS-CoV-2 trends. We aimed to investigate whether this approach could be applied to influenza A/B viruses and respiratory syncytial virus (RSV) in Marseille, southern France. Wastewater concentrations of SARS-CoV-2, influenza A/B viruses, and RSV in Marseille were monitored by qPCR between January 2021 and October 2024. These concentrations were compared with the diagnosis numbers for the three viruses collected at public hospitals in Marseille, using cross-correlation analyses. The Granger causality test was used to determine whether wastewater concentrations can predict the number of clinical cases. SARS-CoV-2 and influenza virus concentrations in wastewater preceded the rise in the incidence of patient diagnoses by a lag of five days and nine/ten days, respectively. In contrast, for RSV, the rise in incidence of clinical cases preceded that of wastewater concentrations. We conclude that wastewater-based epidemiology is a powerful tool to monitor the level of circulation of these viruses independently of tests carried out on people. It enables earlier alerts than monitoring patients for SARS-CoV-2 and influenza symptoms. However, for RSV, it does not provide an early warning, and clinical data-based surveillance appears to be more suitable.

## 1. Introduction

Wastewater-based epidemiology (WBE) was extensively and successfully used during the COVID-19 pandemics. Thanks to the detection and quantification of severe acute respiratory syndrome coronavirus 2 (SARS-CoV-2) RNA in wastewater samples, virus trends could be comprehensively monitored in real time, making it possible to estimate population-wide SARS-CoV-2 prevalence. Such surveillance provides a global picture of viral dynamics and transmission and provides an early warning for the emergence of new epidemics, which can be useful to implement preventive measures. For hospitals, this could contribute towards planning the organisation of medical and paramedical staff and of laboratory technicians. WBE using quantitative real-time PCR reflects the level of virus circulation within cities, and this approach appears to be robust and highly cost-effective compared with the traditional approach, which is based on clinical data that do not take into account undiagnosed, presymptomatic, and asymptomatic cases and only provide a partial picture of viral circulation. Asymptomatic cases constitute at least one-third of SARS-CoV-2 infections and the presymptomatic period lasts approximately two to five days [1,2]. It has been previously demonstrated that SARS-CoV-2 RNA concentrations in sludge were correlated with the number of new diagnoses and were detectable from zero to two days before positive clinical test results, and from one to four days before local hospital admissions [3,4].

It has been previously shown that nanopore sequencing of isothermal rapid viral amplification allowed not only the detection but also the monitoring of mutations of SARS-CoV-2, influenza A, human adenovirus, and human coronavirus for 96 samples concomitantly [5]. It should be noted that viral epidemiology based on wastewater samples has also been developed for non-respiratory viruses such as the hepatitis A virus, for which HAV RNA concentrations were correlated with rates of hepatitis A cases [6]. A larger study carried out in Italy targeted human SARS-CoV2 but also adenovirus, norovirus genogroup II, and enterovirus through reverse transcription qPCR demonstrated temporal–local viral distribution, especially with the demonstration of seasonal peaks for human adenovirus and enterovirus and a shift from an epidemic to an endemic pattern for SARS-CoV2 in the region under investigation, interpreted as the spreading of new variants [7].

In contrast to SARS-CoV-2, limited research has been conducted on other respiratory viruses. For respiratory syncytial virus (RSV) and influenza A/B virus, some studies have reported correlations between wastewater concentrations and clinical cases. However, these associations were not consistently observed across all investigations [8,9,10,11,12,13]. While the usefulness of wastewater surveillance as an early warning system has been well established for SARS-CoV-2, findings remain inconsistent for RSV and influenza [14,15].

Our work focused on SARS-CoV2, RSV, and influenza A/B virus, which are some of the main causes of upper and lower respiratory infections and cause a substantial number of hospitalisations every year at our institution, which encompasses public and university hospitals in Marseille, southern France [16]. Alongside SARS-CoV-2 concentrations, we quantified those for RSV and influenza A/B virus in Marseille wastewater samples collected over a three-year period, and conducted a correlation analysis with corresponding clinical data collected for our institution. *In fine*, the objective was to assess whether simple and inexpensive methods could be used to assess the quantitative epidemiology of the three major respiratory viruses in order to guide decision-makers in launching the recruitment of reinforcements (laboratory technicians and healthcare personnel) in order to mitigate the annual overrun of care capacities.

## 2. Materials and Methods

### 2.1. Wastewater Sampling

Each day, two wastewater samples were collected from two distinct sewer networks in Marseille, a city with one million inhabitants. The 24 h composite samples were collected by pooling multiple and regular grab samples at a specified frequency. This collection method is considered as more sensitive and more representative of community faecal contributions than grab samples [17,18]. The separate network (noted as RS) drains the majority of wastewater in Marseille, including that of the city’s hospitals. The combined network (noted as RU), carries both rainwater and wastewater and drains the city centre of Marseille, as previously explained [19]. The distances of the furthest points from the collection point for the separate network and the unitary network are respectively 24 and 12 km. These samples were collected by the SERAMM (Marseille Metropole Sanitation Department) by two independent “ASP-Station 2000 RPS20B” vacuum samplers (Endress Hauser, Huningue, France). This sampler collected wastewater from over a 24 h period, filling a 20-litre refrigerated flask every 24 h. Samples were transferred every day on ice to the nuclear, radiological, biological, chemical laboratory (NRBC unit) of the Marseille Fire Brigade (BMPM) and were treated within one hour of collection. Large particles were removed from the samples using a Millex sterile syringe filter with a pore size of 5 µm (Merck Millipore, Molsheim, France). A 20 mL aliquot of each sample was filtered. Collection dates began in January 2021 for SARS-CoV-2, November 2022 for influenza viruses, and October 2022 for RSV.

### 2.2. DNA/RNA Extraction and RT-PCR Screening of SARS-CoV-2, Influenza A/B Viruses, and RSV for Wastewater Samples

Viral RNA was extracted using the eGENE-UP^®^ Magnetic silica, Lysis and RNA Purification kit (bioMérieux, Marcy l’Etoile, France) on 1 mL of each wastewater sample to obtain a final eluate volume of 100 µL. A negative control was prepared with RNAse-free water, following the same protocol as for the samples.

Screening by real-time reverse transcription (RT) PCR (qPCR) of SARS-CoV-2 RNA in wastewater samples collected between 1 January 2021 and 26 November 2022 was performed using the QuantStudio 5 RT-PCR system (Thermo Fisher Scientific, Carlsbad, CA, USA) with the Bio-T kit^®^ TriStar Covid-19 (Biosellal, Dardilly, France). The influenza virus was included in this screening from 27 November 2022 using the MIC qPCR system (Bio Molecular Systems, Upper Coomera, Australia) with the Bio-T kit^®^ Environmental Covid & Flu (Biosellal, Dardilly, France), according to the supplier’s protocols. Finally, screening of RSV was added from 28 October 2022 using the QuantStudio 5 RT-PCR system (Thermo Fisher Scientific, Carlsbad, CA, USA) with the Premium Dx^®^ ColdPlex Covid & Flu & RSV (Biosellal, Dardilly, France), following the supplier’s protocols. A negative and a positive control were added in each run of PCR consisting respectively in distilled water and culture supernatant. The virus concentrations were quantified using a standard curve obtained by serial dilutions of standards provided by the qPCR kit suppliers. The limits of detection for SARS-CoV-2, influenza virus, and RSV determined by the manufacturer are 333, 667, and 154 genome copies per mL, respectively.

### 2.3. DNA/RNA Extraction and qPCR Diagnosis of SARS-CoV-2, Influenza A/B Viruses, and RSV for Clinical Samples

The numbers of qPCR tests performed which were positive for the diagnoses of infections with SARS-CoV-2, influenza A/B viruses, and RSV were collected retrospectively from the information system of our clinical microbiology laboratory of public and university hospitals of Marseille (AP-HM). These diagnoses had been performed on respiratory samples collected from patients between January 2021 and October 2024 and sent to our laboratory as part of routine clinical testing. The collection and analysis of these data were registered on the AP-HM Health Data Access Portal under No. PADS25-147 and were approved by the AP-HM Ethics and Scientific Committee (Number CSE25-203). Viral diagnoses had been carried out using three different techniques depending on physicians’ prescriptions and requested timescale for results. These included: (1) the Fast Track Diagnosis Respiratory pathogens 21 assay (Fast Track Diagnosis, Luxembourg), which simultaneously detects 20 viruses and one bacterium; (2) the FilmArray Respiratory panel 2.1 plus assay (RP2.1plus) (bioMérieux, Marcy-l’Etoile, France), which simultaneously detects 19 viruses and four bacteria; and (3) the GeneXpert Xpert Xpress CoV-2/Flu/RSV plus quadruplex assay (Cepheid, Sunnyvale, CA, USA). For the Fast Track Diagnosis Respiratory pathogens 21 assay, DNA/RNA was extracted from respiratory swabs using the KingFisher Flex 105 system (Thermo Fisher Scientific, Ecublens, Switzerland) according to the manufacturer’s instructions.

### 2.4. Comparison of Wastewater and Clinical Data

Data obtained here from the sewage samples were compared to those obtained from the patients’ respiratory samples. Concentrations (genome copies number/mL) of each virus from the RS and RU were calculated and adjusted according to the respective population of these two network areas when combined, as previously described [19]. A seven-day sliding average was determined and results were correlated with seven-day sliding average of positive diagnoses of SARS-CoV-2, influenza virus, and RSV infections.

### 2.5. Statistical Analysis

After normalization, the cointegration analyses of time series data were performed using Autoregressive Distributed Lag (ARDL) models [20]. These models are particularly suited for our data as they can accommodate series with different integration orders, which is common in exploratory epidemiological time series analysis, as long as none are I(2), even if they are mixed. The ARDL framework is also the foundational structure for the bounds testing approach to cointegration and naturally lends itself to testing for causal relationships. To explore the specific predictive relationships within this cointegrated system, we chose the Granger causality test [16]. While other multivariate time-series models, such as Vector Autoregression (VAR) or Transfer Function Models, are powerful for modelling complex, multi-directional feedback loops, our primary objective was an exploratory assessment of whether changes in sewage viral concentrations precede changes in clinical case numbers. The Granger causality test is a focused, robust hypothesis test designed specifically for this purpose. By successively considering each pathogen’s viral concentration first as a dependent then as an independent variable, it allowed us to directly test the core hypothesis of the study: whether the sewage data could serve as a predictive early warning signal for human cases. This approach, built upon the foundation of our cointegration analysis, provided a clear and interpretable framework for assessing the temporal precedence of one series over another. A crucial aspect of inferring causal relationships from time series is ensuring that the sampling resolution is adequate to capture the underlying temporal dynamics. The time series data for this study consisted of daily measurements of viral concentrations in sewage and daily reported clinical case incidences. This daily resolution aligns with the expected biological and epidemiological time scales of viral transmission and disease progression, which are known to occur over a period of days, depending on the agent’s latent period. Cross-correlation analyses were first used to inform the selection of optimal lags between the time series, ensuring that the model’s temporal structure was well-specified. The lag values are critical as they indicate if the temporal delay of the causal influence is significantly longer than our daily sampling interval. Had the causal relationship been instantaneous or occurred within a sub-daily time frame, our chosen sampling frequency would have been insufficient to capture the temporal precedence required for a valid Granger causality assessment. All statistical analyses were conducted using R version 4.4.1.

## 3. Results

### 3.1. SARS-CoV2 (Figure 1 and Figure 2, Supplementary Data)

**Figure 1:**
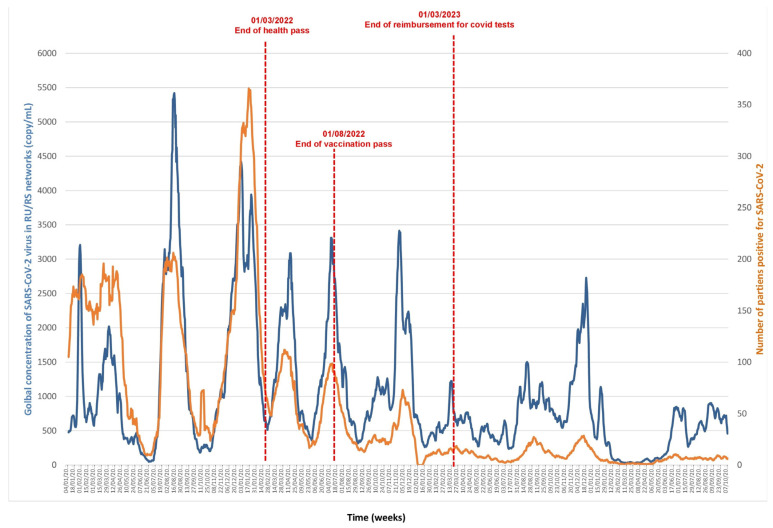
Monitoring SARS-CoV-2 RNA concentration in Marseille wastewater (blue line) and number of positive diagnoses of SARS-CoV-2 in patients (orange line) from January 2021 to October 2024.

**Figure 2:**
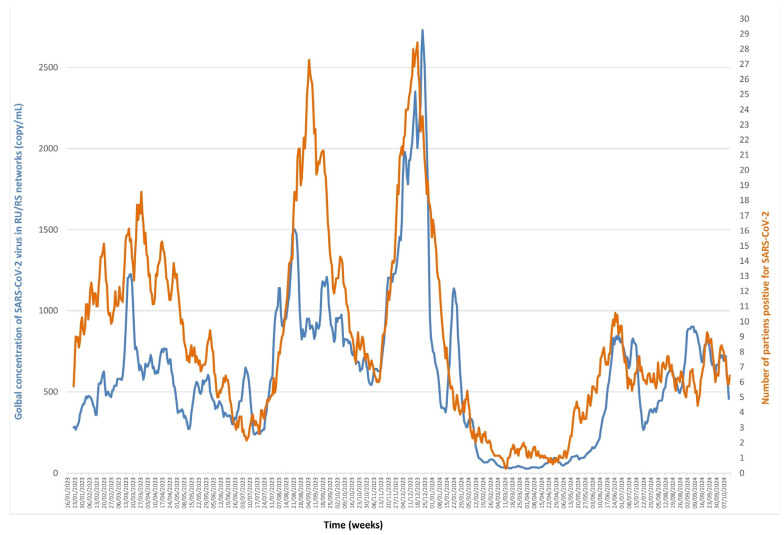
Monitoring SARS-CoV-2 RNA concentration in Marseille wastewater (blue line) and number of positive diagnoses of SARS-CoV-2 in patients (orange line) from January 2023 to October 2024.

Wastewater concentration varied from undetectable to a maximum of 7674 copies/mL. The cross-correlation between wastewater concentrations and the number of positive diagnoses shows a maximal correlation at 0.724 with a lag of five days, which is significantly longer than our daily sampling interval. This indicates that trends in virus concentration in wastewater precedes those of the numbers of positive diagnoses. The ARDL analyses, with viral concentration as both dependent and independent variables, shows a cointegration between the series, as indicated by a significant Wald bounds F-test for no cointegration (F = 15.231, *p* = 7.948 × 10^−5^, and F = 17.061, *p* = 10^−6^). Additionally, the Error Correction Terms were significant and negative (estimate = −0.107, t = −5.159, *p* = 2.85 × 10^−7^, and estimate = −0.112, t = −5.086, *p* = 4.18 × 10^−7^), confirming the validity of the cointegration relationship. The Granger causality test indicated that the viral concentration series has a predictive power over the case positivity series (F = 8.000, *p* = 1.392 × 10^−10^). Furthermore, the test also shows that case positivity can be used to forecast viral concentrations (F = 3.365, *p* = 0.0015). The level of correlation also varies according to the period studied. The number of positive diagnoses dropped drastically after each change in public health measures targeting the COVID-19 epidemic, first with the end of the “health pass”, then with the end of the “vaccination pass”, and finally with the end of reimbursement of COVID-19 tests by the public health insurance system (Figure 1). However, once the number of tests performed was reduced, the curves continued to be correlated (Figure 2).

### 3.2. Influenza A/B Viruses (Figure 3, Supplementary Data)

**Figure 3:**
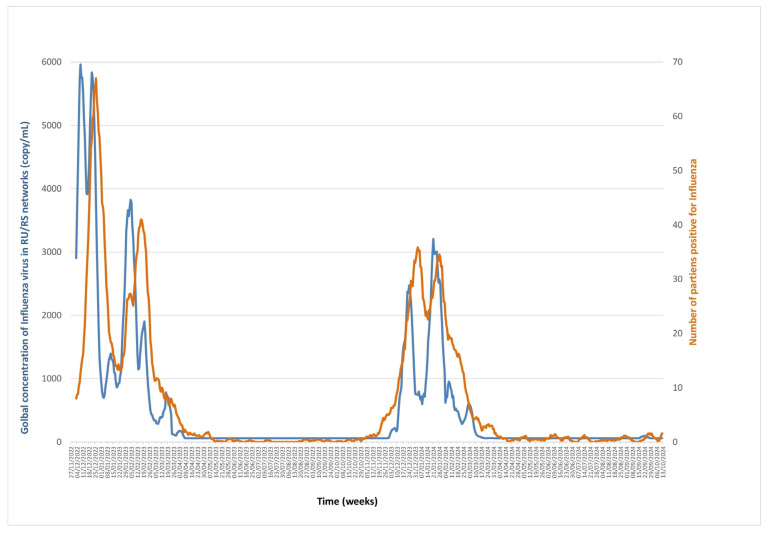
Monitoring influenza virus RNA concentrations in Marseille wastewater (blue line) and number of positive diagnoses of influenza viruses in patients (orange line) from November 2022 to October 2024.

Influenza virus wastewater concentrations were measured from November 2022 to October 2024 and varied from undetectable to 7586 copies/mL. The cross-correlation between the two series showed maximal correlations of 0.803 and 0.804 at lags of nine and ten days, respectively, with wastewater virus concentrations preceding positive diagnoses by nine to ten days. ARDL analyses of virus concentration as both dependent and independent variables indicated the presence of a cointegrating relationship between the series with a significant Wald bounds F-test for no cointegration (F = 26.61, *p* = 10^−6^, and F = 52.196, *p* = 10^−6^), and significant and negative Error Correction Terms (estimate = −0.138, t = −6.705, *p* = 4.31 × 10^−11^, and estimate = −0.208, t = −9.475, *p* < 2 × 10^−16^), confirming the validity of the cointegration relationship. The Granger causality test showed that wastewater virus concentrations forecasted numbers of positive diagnoses (F = 3.062, *p* = 0.0058), and that numbers of positive diagnoses also forecasted wastewater virus concentrations (F = 12.171, *p* < 2.2 × 10^−16^) (Figure 3).

### 3.3. RSV (Figure 4, Supplementary Data)

RSV wastewater concentrations were assessed from October 2022 to September 2024 and ranged from undetectable to 46,618 copies/mL. The cross-correlation between the two series showed a maximal correlation of 0.693 and 0.692 for a lag of eight and nine days, the trends of the number of positive diagnoses preceding those of wastewater virus concentration. A second optimal correlation could be observed for a lag of one day (0.691). The presence of this second optimal correlation may be explained by the presence in the series of two seasonal peaks with different lags. A first analysis with concentration as the dependent variable showed a cointegration of the series, supported by a significant Wald bounds F-test for no cointegration (F = 38.423, *p* = 10^−6^). This was further confirmed by a significant and negative Error Correction Term (estimate = −0.230, t = −8.741, *p* < 2.2 × 10^−16^), validating the cointegration relationship. The Granger causality test confirms that numbers of positive diagnoses were predictive of wastewater virus concentrations (F = 21.966, *p* < 2.2 × 10^−16^). However, analysis of the wastewater virus concentration as an independent variable showed no cointegration between the series (F = 4.5179, *p* = 0.1212), indicating no predictive relationship in this direction.

## 4. Discussion

Data on the simultaneous monitoring of SARS-CoV-2, RSV, and influenza virus concentrations in wastewater remain limited, and existing findings are not consistently aligned [12,14].

In this study, we showed that rises in SARS-CoV2 and influenza virus concentration in wastewater preceded rises in the number of positive diagnoses with a lag of five days and nine to ten days, respectively. However, for RSV, the opposite was observed, as the rise in the number of positive diagnoses preceded the rise in wastewater concentrations. Our laboratory, integrated within the University Hospital (CHU), serves as the central facility for microbiological analyses across all public hospitals in the city. The patient population predominantly originates from the Marseille metropolitan area, although a small proportion may reside outside the city. 

Previous studies demonstrated that concentrations of SARS-CoV2 and influenza virus RNA in wastewater settled solids were strongly positively correlated with positivity rates of clinical cases. Numerous studies previously showed the usefulness of SARS-CoV-2 environmental surveillance for monitoring viral transmission, epidemic waves, and tracking emerging variants, as the wastewater signal increased on average one to two weeks before clinical cases [21,22,23,24,25]. Furthermore, the quantification and even subtyping in some studies of influenza viruses in wastewater was previously successfully performed and made it possible to forecast flu outbreaks between seven and seventeen days earlier than traditional surveillance based on clinical diagnoses [26,27]. In this study, it is extremely interesting to note that such an association between SARS-CoV-2 concentrations in wastewater and the number of clinical positive diagnoses persisted independently of the number of tests carried out, demonstrating the robustness of this association.

In contrast to SARS-CoV-2 and influenza viruses, fewer studies have been conducted on RSV in wastewater. Notably, Zambrana et al. showed that the predominant RSV subtype was the same in wastewater and clinical samples [28]. Beyond this, there are many fewer studies on RSV concentrations in wastewater and their correlation with the incidence of clinical cases than for SARS-CoV-2 and influenza viruses, with no standardisation between the studies. RSV RNA concentrations in wastewater solids were measured to range from undetectable to 10^7^ copies per gram and were correlated with RSV incidence in patients [15,27,29]. Regarding the predictive value of RSV wastewater concentrations on the onset of RSV outbreaks, studies are not consistent. Zulli et al. showed in the United States that in three of fourteen states studied, the rise in wastewater virus concentrations preceded the onset of the outbreak [15], in contrast to what we found here. However, in half of the states, the rise in wastewater concentrations occurred after the outbreak onset, which is consistent with our findings. In another study carried out in Hamilton and Ottawa, Canada, a 12-day lag was found between RSV wastewater concentrations (normalised per gram of wastewater solids) and paediatric RSV diagnoses (Spearman’s ρ  =  0.90) [26].

Differences in the timing of the onset of RSV outbreaks as observed based on wastewater RSV concentrations or clinical data may be due to various factors. First, wastewater surveillance approaches were not all standardised in terms of sampling mode, sampling frequency, and types of analyses performed. In particular, regarding sample types, some teams analysed settled solids while others (including us) focused on the liquid fraction of wastewater. Indeed, it has been previously shown that up to 26% of enveloped viruses and 6% of non-enveloped viruses are adsorbed onto the solid wastewater fraction [30]. Thus, although all three viruses studied here are enveloped, the type of wastewater sample used might contribute to differences between studies regarding RSV. Noteworthy, the centrifugation step to remove large particles used in our protocol may have an impact on the virus recovery rate by removing concomitantly viral particles settled with the pellet [31]. It has been previously demonstrated that viruses partitioned differently between liquid and solid phases [32]. For example, SARS-CoV2 attached more strongly to solid particles compared with Bocaviruses [33] and viral concentration has been assessed as three to four times higher in solid than in liquid phase of wastewater for SARSCoV2, Rhinovirus and RSV [32].

Also, the PCR methods were not standardised and may notably differ by their detection thresholds.

Moreover, wastewater samples are composed of a complex mixture and the normalization of the viral concentration allows to take into account changes due to parameters such as precipitation or the size of the population over time. A normalization of results by using the proportion of the virus of interest upon a stable and abundant biomarker present in wastewater can be used for standardize the results between studies. Pepper mild mottle virus (PMMoV), cross-assembly phage (crAssphage), Bacteroides rRNA, 18 S rRNA, creatinine, 5-hydroxyindoleacetic acid, and caffeine, as well as ammonium (derived from urine) or *E. coli*, have already been used in several studies. However, all studies are not congruent about the interest of the use of these biomarkers and the biomarkers are not standardized.

Zhan et al. showed that normalized SARS-CoV2 levels by PMMoV was better correlated with COVID19 cases [34]. While Bridgette Hughes et al. [29] demonstrated that an increase in RSV RNA was associated with an increase in RSV positivity rate and that the results were unchanged if the concentrations were normalized by PMMoV. Another work showed that not all biomarkers were equivalent for all viruses; for example, influenza A concentrations normalized with *Escherichia coli*, and total coliforms were correlated with clinical cases while normalized SARS-CoV2 concentrations with PMMoV were not correlated with clinical cases [35].

Second, the natural progression of RSV infections and the dynamics of RSV outbreaks have characteristics that differ from those for SARS-CoV-2 and influenza viruses, which may impact differently on RSV detection in wastewater and may result in the differences in the lag times relative to the rise in clinical diagnoses. The presymptomatic period, which may allow earlier signals to be detected based on wastewater testing than based on clinical diagnoses, is fairly similar across the three viruses, lasting approximately three days [36,37,38,39]. Thus, this should not play a role in the differences observed in the present study. However, the proportion of asymptomatic cases varies significantly across the three viruses. Thus, it was estimated that the proportion of asymptomatic RSV infections is <5% in adults and approximately 10% in children [40,41,42], which is much lower than for influenza virus infections, with proportions ranging% from 19 to 85% [43,44,45], and for SARS-CoV-2, with proportions ranging from 30% to 70%, with differences according to age and the viral variant [46,47,48,49].

In addition, going back to basics, environmental surveillance is primarily feasible because viruses are excreted in faeces or urine. While faecal excretion of SARS-CoV-2 and influenza viruses is well documented, data on RSV remain scarce. Shedding in faeces has been reported in only 10% to 14% of RSV infections [50,51] compared with 36% 50% of influenza virus infections [51,52] and 30% to 75% of SARS-CoV-2 infections [53]. Concentrations in faeces were estimated to be 10^2^–10^7^ copies/mL for SARS-CoV-2 and 10^4^ copies/mL for influenza viruses, with a mean shedding duration of three weeks [54,55,56,57,58]. Unfortunately, there is no such data to our knowledge on RSV [59,60]. Moreover, while viral shedding in urine may contribute to the overall viral load in wastewater for SARS-CoV-2 and influenza viruses, which are both excreted in urine, in 58% and <5% of cases, respectively, it remains unclear whether RSV is excreted in urine [51,61].

Pathogen shedding rate per person per day has also to be considered. The SARSCoV2 shedding has been assessed to 5.3 × 10^7^–9.61 × 10^11^ gene copies per individual per day [62]. Unfortunately, there are no such existing data to our knowledge, for RSV and influenza.

Finally, differences related to the ages of infected patients may explain differences between RSV and the two other viruses. Indeed, RSV load is particularly high in children under the age of five [63]. In addition, among children under one year old, the median viral load in faeces is 1.88 time higher than in children between the ages of one and five, 2.84 times higher than in children between the ages of five and fifteen, 4.86 times higher than in young people between 15 and 40, and 1.26 time higher than in adults over the age of 40. This was observed in absence of differences in viral shedding kinetics according to age. Hence, taken together, the low frequency of RSV excretion in stools and the fact that the greatest shedders are infants under the age of one who generally still wear nappies may explain the differences observed between kinetics of wastewater concentrations for RSV on the one hand and for SARS-CoV-2 and influenza viruses on the other hand.

## 5. Conclusions

In conclusion, in our centre, wastewater-based surveillance was a scalable and robust tool to monitor SARS-CoV-2, influenza viruses, and RSV outbreaks. Wastewater concentrations provided earlier warnings than patient diagnoses for SARS-CoV-2 and influenza virus outbreaks with respective lag times of five days and nine/ten days, although not for RSV outbreaks. Our results can be applied to different situations and different sewer system configurations, from the smallest with aircraft lavatories, nursing homes, university campus, or partial municipal sewer system to the biggest with sewer systems corresponding to more than 5 million persons in a mega city such as Hong Kong [25,64,65,66,67].

More studies by different teams are warranted for RSV wastewater surveillance especially by analysing solid fractions of wastewater, considering the high levels of incidence of infections during outbreaks and the considerable associated morbidity and mortality, in addition to the recent implementation of efficient new preventive measures [68].

## Figures and Tables

**Figure 4:**
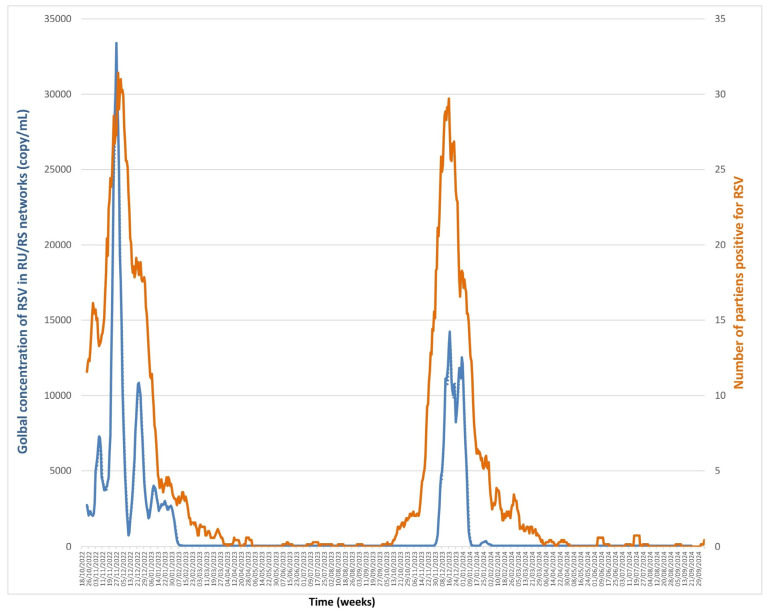
Monitoring respiratory syncytial virus (RSV) RNA concentrations in Marseille wastewater (blue line) and number of positive diagnoses of RSV in patients (orange line) from October 2022 to September 2024.

## Data Availability

The original contributions presented in the study are included in the article/Supplementary Materials. Further inquiries can be directed to the corresponding authors.

## Supplementary Materials

The following supporting information can be downloaded at: https://www.mdpi.com/article/10.3390/microorganisms13102268/s1, Supplementary Data: Raw data of wastewater concentrations and number of diagnoses per virus and per date.
